# Smoking increases the risk of early postoperative infection after elective total hip arthroplasty: Evidence from a Nationwide Japanese database

**DOI:** 10.1007/s00264-026-06747-w

**Published:** 2026-02-11

**Authors:** Hidetatsu Tanaka, Kunio Tarasawa, Yu Mori, Hideki Fukuchi, Kiyohide Fushimi, Toshimi Aizawa, Kenji Fujimori

**Affiliations:** 1 Department of Orthopaedic Surgery, Tohoku University Graduate School of Medicine, Sendai, Japan; 2 Department of Medical Information Technology Center, Tohoku University Hospital, Sendai, Japan; 3 Department of Health Policy and Informatics, Institute of Science Tokyo, Tokyo, Japan; 4 Tohoku University, Sendai, Japan

**Keywords:** Total hip arthroplasty, Smoking, Complication, Diagnosis Procedure Combination, Propensity score analysis

## Abstract

**Purpose:**

Smoking is a potentially modifiable risk factor for adverse outcomes after total hip arthroplasty (THA), but evidence on early postoperative complications in Asian populations remains limited. This study examined the association between smoking and early postoperative complications after elective THA using a nationwide inpatient database in Japan.

**Methods:**

This retrospective cohort study analysed data from the Japanese Diagnosis Procedure Combination (DPC) database between December 2011 and March 2023. Patients undergoing elective primary THA for osteoarthritis, osteonecrosis of the femoral head, or rheumatoid arthritis were included. Smoking status was identified using administrative codes. One-to-one propensity score matching was used to balance baseline characteristics between smokers and non-smokers. Primary outcomes were early postoperative surgical complications, medical complications, and in-hospital mortality. Dose-dependent effects were assessed using the Brinkman Index, with heavy smoking defined as ≥ 600.

**Results:**

After propensity score matching, 52,551 patients were included in each group. Smoking was associated with a higher risk of postoperative infection (odds ratio [OR] 1.31; 95% confidence interval [CI] 1.15–1.49; *p* < 0.001) and a lower likelihood of blood transfusion (OR 0.83; 95% CI 0.80–0.85; *p* < 0.001). No significant associations were observed with dislocation, periprosthetic fracture, wound dehiscence, reoperation, major medical complications, or in-hospital mortality. Heavy smoking (Brinkman Index ≥ 600) was not associated with postoperative complications.

**Conclusions:**

Smoking was associated with an increased risk of early postoperative infection following elective THA, but not with other major complications or in-hospital mortality. Smoking cessation should be considered an important component of perioperative optimisation.

## Introduction

Total hip arthroplasty (THA) has been described as one of the most successful surgical procedures of the century, providing substantial pain relief and functional recovery for patients with degenerative hip disease [1]. Complications following THA can be devastating for patients and impose a substantial economic burden. In addition to age-related comorbidities, several potentially modifiable risk factors—such as diabetes, obesity, and alcohol consumption—have been identified and may be targeted to reduce postoperative complications and improve surgical outcomes [2–5]. Smoking represents another important modifiable risk factor that has been consistently associated with an increased risk of postoperative complications following THA [4, 6–20].

Several large retrospective studies have demonstrated that smoking is associated with an increased risk of infection [13–15, 20], wound-related complications [20, 21], hospital readmission [17, 20], and early revision following THA [13, 19]. Furthermore, smoking has also been associated with a higher risk of medical complications, including cardiac arrest, urinary tract infection, sepsis, acute renal failure, discharge to a skilled nursing facility, and mortality [4, 19]. Prior retrospective studies, primarily based on small to moderate-sized cohorts, have reported an association between smoking and an increased risk of infection [12], readmission [16], early revision following THA [12], and pneumonia [10]. However, not all studies have demonstrated consistent associations between smoking and adverse outcomes after THA. Some investigations reported no significant differences in overall complication rates or postoperative clinical scores between smokers and non-smokers [8]. In addition, smoking was not associated with an increased risk of dislocation or prolonged length of hospital stay in certain retrospective analyses [12]. Furthermore, several reports failed to identify a significant association between smoking and postoperative infection [12, 17], suggesting that the impact of smoking on specific complications after THA may vary across study populations and methodological designs.

Although smoking rates in Japan have declined over recent decades, smoking remains a persistent public health challenge [22]. To date, no comprehensive nationwide study has evaluated the association between smoking and perioperative outcomes of THA in Japanese patients. The purpose of this study was to investigate whether smoking is associated with early postoperative complications following elective THA using the Japanese Diagnosis Procedure Combination (DPC) nationwide inpatient database. Specifically, we examined the associations between smoking status and (1) hip- and surgery-related complications, (2) medical complications, and (3) in-hospital mortality, while accounting for baseline differences between the study groups.

## Material and methods

### Study design and setting

This retrospective cohort study was conducted using data from the Japanese Diagnosis Procedure Combination (DPC) database and adhered to the principles of the Declaration of Helsinki. The study protocol was approved by the Institutional Review Boards. The study period spanned from December 2011 to March 2023 and encompassed data from approximately 1,100 hospitals participating in the nationwide DPC system in Japan. The DPC database contains anonymized patient-level information, including demographic characteristics, diagnoses coded according to the International Classification of Diseases, Tenth Revision (ICD-10), dates of hospital admission and discharge, details of surgical and nonsurgical procedures, pre-existing comorbidities, and in-hospital complications.

### Participants and data selection

This study included patients who underwent THA for hip osteoarthritis (ICD-10 codes M160–M169), osteonecrosis of the femoral head (M8705, M8715, M8725, M8735, M8785, and M8795), or rheumatoid arthritis (M0695, M0690, M0685, M0605, M0595, M0585, M0586, and M1315). Patients undergoing THA for trauma-related indications, including proximal femoral or acetabular fractures, were excluded. In addition, cases with missing data for key covariates, such as body mass index or smoking status, were excluded. Between December 2011 and March 2023, 309,347 eligible patients were identified. The analysis was restricted to resource-intensive cases defined by the DPC system, reflecting real-world THA practice in Japan.

Propensity score (PS) matching was performed in a 1:1 ratio using age, sex, body mass index (BMI), and the Charlson Comorbidity Index (CCI) as covariates, with smoking status defined as a binary exposure variable. The discriminative ability of the PS model was evaluated using the C-statistic. Nearest-neighbor matching without replacement was conducted with a caliper width of 0.2 times the standard deviation of the PS. After matching, 52,551 patients were included in each of the smoking and non-smoking groups. The C-statistic was 0.813, and all covariates achieved standardized mean differences (SMDs) of < 0.1, indicating adequate covariate balance between the groups (Table 1).

**Table 1:** Baseline demographic data according to smoking status

		Before PS matching			After PS matching	
	Smoking	Non-smoking	*P*-value	Smoking	Non-smoking	SMD
n	57044	252303		52551	52551	
Age	62.6 ± 11.2	68.4 ± 10.7	< 0.001※	63.5 ± 11.0	62.9 ± 11.6	0.055
Gender (%)						
Men	27495 (48.2)	25893 (10.3)	< 0.001	23005 (43.8)	22550 (42.9)	0.017
Women	29549 (51.8)	226410 (89.7)		29546 (56.2)	30001 (57.1)	
BMI	24.5 ± 4.3	24.0 ± 4.1	< 0.001※	24.4 ± 4.3	24.5 ± 4.2	0.019
Uni-rateral THA	55248 (96.9)	245428 (97.3)	< 0.001	50916 (96.9)	50937 (96.9)	0.002
Diagnosis for THA (%)						
OA	45496 (79.8)	232795 (92.3)	< 0.001	43488 (82.7)	44585 (84.8)	0.068
ONFH	10846 (19.0)	15975 (6.3)		8397 (16.0)	7335 (14.0)	
RA	702 (1.23)	3533 (1.4)		666 (1.3)	631 (1.2)	
CCI	0.56 ± 0.93	0.45 ± 0.80	< 0.001	0.55 ± 0.91	0.53 ± 0.91	0.002
Length of hospital stay	24.6 ± 14.6	26.9 ± 16.2	< 0.001	24.7 ± 14.6	25.7 ± 15.6	0.063
Cement	10,329 (18.1)	44,770 (17.7)	0.041	9615 (18.3)	8594 (16.4)	0.051
CAOS	16,482 (28.9)	70,332 (27.9)	< 0.001	15,260 (29.0)	14,794 (28.2)	0.020
Post-operative Anticoagulate agent	41,454 (72.7)	190,229 (75.4)	< 0.001	38,265 (72.8)	39,274 (74.7)	0.044

Age, BMI, CCI, and length of hospital stay are shown as mean ± standard deviation

P-values of < 0.001 are considered significant by the Student-t test andχ2 test

BMI: Boby Mass Index, THA; Total Hip Arthroplasty, OA: Osteoarthritis, ONFH; Osteonecrosis of Femoral Head, RA; Rhumatoid Arthritis, CCI; Charlson Comobidity Index, CAOS; Computer Assisted Orthopaedic Surgery SMD; Standardized Mean Difference

※ Student-t test

**Fig. 1:**
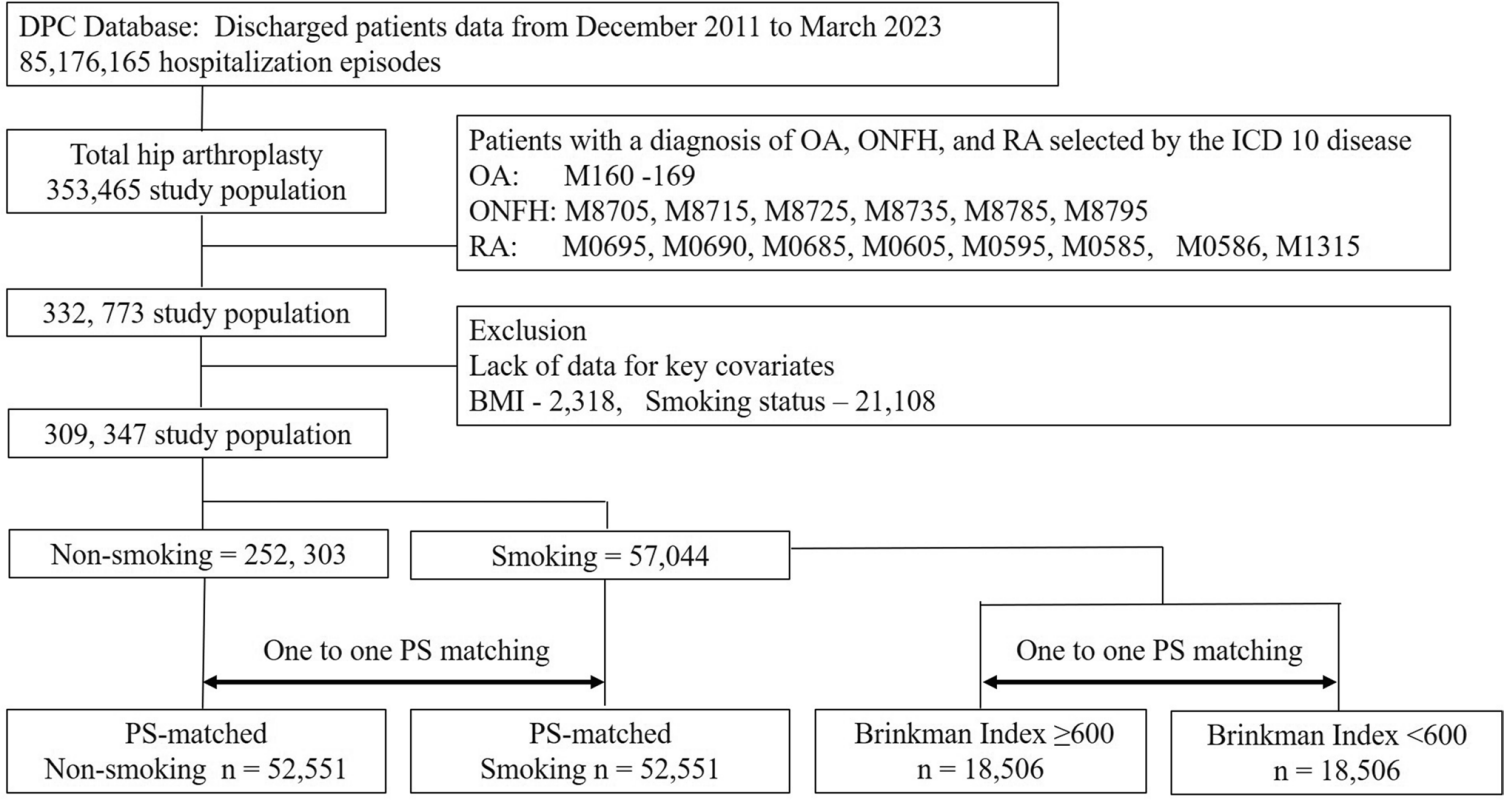
Study flow chart

Additionally, to compare the relationship between smoking exposure and postoperative complications, we conducted 1:1 PS matching stratified by Brinkman Index (≥ 600 vs < 600), calculated as the number of cigarettes smoked per day multiplied by the number of smoking years. Heavy smoking was defined as a Brinkman Index ≥ 600, consistent with definitions used in previous orthopaedic clinical studies [23, 24]. A flowchart of study inclusion is shown in Fig. 1.

### Exposure and outcomes

Patients were classified into smoking and non-smoking groups based on perioperative medication codes recorded on the day of surgery. Primary surgical outcomes included postoperative dislocation, surgical site infection, periprosthetic fracture, wound dehiscence, transfusion, and reoperation. Medical complications comprised hospital-acquired pneumonia, deep vein thrombosis (DVT), pulmonary embolism (PE), cardiac events, cerebrovascular events, acute renal failure, and in-hospital mortality.

### Statistical analysis

Categorical variables were compared using the chi-squared test, and continuous variables were analyzed using Student’s t-test. Univariate and multivariate logistic regression analyses were conducted to evaluate the association between smoking status and postoperative complications, with adjustment for relevant covariates. Continuous data are presented as mean ± standard deviation (SD), and effect estimates are reported as odds ratios (ORs) with 95% confidence intervals (CIs). Statistical significance was defined as *p* < 0.001, given the large sample size of this nationwide study, and a standardized mean difference (SMD) of < 0.1 was considered indicative of adequate covariate balance. All statistical analyses were performed using JMP version 18.0 (SAS Institute, Cary, NC, USA).

## Results

Smoking was associated with a significantly increased risk of infection (OR, 1.25; 95% CI 1.10–1.42; *p* < 0.001), and a significantly lower risk of blood transfusion (OR, 0.82; 95% CI, 0.80–0.84; *p* < 0.001). No significant association was found between smoking and dislocation (OR, 0.87; 95% CI, 0.72–1.05; *p* = 0.154), periprosthetic fracture (OR, 1.13; 95% CI, 0.90–1.40; *p* = 0.291), wound dehiscence (OR, 1.14; 95% CI, 0.82–1.59; *p* = 0.438), reoperation (OR, 1.16; 95% CI, 1.01–1.34; *p* = 0.036), hospital-acquired pneumonia (OR, 1.02; 95% CI, 0.75–1.40; *p* = 0.901), DVT (OR, 0.96; 95% CI, 0.90–1.01; *p* = 0.114), PE (OR, 0.92; 95% CI, 0.66–1.27; *p* = 0.610), cardiac events (OR, 1.80; 95% CI, 0.66–4.88; *p* = 0.239), cerebrovascular events (OR, 0.89; 95% CI, 0.22–3.13; *p* = 0.460), acute renal failure (OR, 1.45; 95% CI, 0.67–3.13; *p* = 0.343), or in-hospital mortality (OR, 0.91; 95% CI, 0.50–1.67; *p* = 0.732) (Table 2).

**Table 2:** Association between smoking and complications

Complications			Univariate analysis			Multivariable analysis		
	n	OR	95% CI	*P-value*	OR	95% CI	χ2 statics	*P-value*
Dislocation	807	1.003	0.873 to 1.151	0.999	0.870	0.718 to 1.054	2.028	0.154
Infection	947	1.307	1.145 to 1.487	< 0.001	1.245	1.097 to 1.424	11.29	< 0.001
Periprosthetic fracture	353	1.193	0.967 to 1.472	0.110	1.125	0.904 to 1.399	1.113	0.291
Wound dehiscence	144	1.182	0.852 to 1.641	0.359	1.142	0.822 to 1.587	0.630	0.438
Transfusion	63,083	0.822	0.801 to 0.842	< 0.001	0.823	0.803 to 0.843	239.4	< 0.001
Reoperation	1601	1.117	1.012 to 1.233	0.030	1.162	1.009 to 1.338	4.385	0.036
Hospital-acquired pneumonia	159	1.039	0.761 to 1.418	0.874	1.02	0.746 to 1.395	0.016	0.901
DVT	5047	0.949	0.897 to 1.005	0.074	0.955	0.903 to 1.011	2.492	0.114
PE	144	0.920	0.663 to 1.276	0.677	0.918	0.662 to 1.274	0.260	0.610
Cardiac event	17	1.834	0.678 to 4.958	0.332	1.798	0.662 to 4.880	1.384	0.239
Cerebrovascular event	173	0.901	0.668 to 1.215	0.543	0.893	0.222 to 3.126	0.547	0.460
Acute renal falure	27	1.455	0.675 to 3.135	0.442	1.447	0.670 to 3.126	0.900	0.343
Mortality during hospitalization	43	0.955	0.525 to 1.736	0.999	0.911	0.496 to 1.670	0.092	0.732

P-values of < 0.001 are considered significant by the χ2 test OR; Odds Ratio, CI; Confidence Interval, DVT; Deep Vein Thrombosis, PE; Pulmonary Embolism

Multivariate logistic regression identified several independent predictors of complications (Table 3). For Infection, male sex (OR, 1.34; 95% CI, 1.18–1.53; *p* < 0.001), higher BMI (OR, 1.06; 95% CI, 1.05–1.07; *p* < 0.001), higher CCI (OR, 1.31; 95% CI, 1.24–1.38; *p* < 0.001), and smoking (OR, 1.31; 95% CI, 1.15–1.49; *p* < 0.001) were independently associated with an increased risk of infection. Age (*p* = 0.817) and unilateral THA (*p* = 0.438) was not significantly associated with infection. For blood transfusion, younger age (OR, 0.99; 95% CI, 0.99–0.99; *p* < 0.001), female sex (OR, 0.87; 95% CI, 0.85–0.90; *p* < 0.001), lower BMI (OR, 0.99; 95% CI, 0.99–1.01; *p* < 0.001), unilateral THA (OR, 0.46; 95% CI, 0.42–0.50; *p* < 0.001), lower CCI (OR, 0.94; 95% CI, 0.93–0.95; *p* < 0.001), and smoking (OR, 0.83; 95% CI, 0.80–0.85; *p* < 0.001) were significantly associated with a lower likelihood of blood transfusion.

**Table 3:** Multivariate logistic analysis of risk factors for complications

	Variable	OR	95% CI	χ2 statics	*P-value*
Infection	Age	0.999	0.994 to 1.005	0.054	0.817
	Gender (Men)	1.340	1.177 to 1.526	19.55	< 0.001
	BMI	1.059	1.045 to 1.074	64.64	< 0.001
	Uni-rateral THA	1.175	0.774 to 1.782	0.601	0.438
	CCI	1.307	1.238 to 1.376	81.98	< 0.001
	Smoking	1.306	1.147 to 1.486	16.50	< 0.001
Transfusion	Age	0.992	0.991 to 0.993	180.2	< 0.001
	Gender (Wemen)	0.873	0.851 to 0.896	110.4	< 0.001
	BMI	0.986	0.989 to 1.014	82.93	< 0.001
	Uni-rateral THA	0.458	0.421 to 0.497	380.0	< 0.001
	CCI	0.939	0.926 to 0.952	81.38	< 0.001
	Smoking	0.825	0.804 to 0.845	231.8	< 0.001

P-values of < 0.001 are considered significant by the χ2 test; OR; Odds Ratio, CI; Confidence Interval, BMI: Boby Mass Index, CCI; Charlson Comobidity Index, DVT; Deep Vein Thrombosis

After one-to-one propensity score matching based on a Brinkman Index ≥ 600, 18,506 patients were included in each group. Baseline characteristics after matching are summarized in Table 4. The C-statistic of the propensity score model was 0.8231, and SMD for all covariates was < 0.1, indicating adequate covariate balance. In both univariate and multivariable logistic regression analyses, no significant associations were identified between a Brinkman Index ≥ 600 and dislocation, infection, periprosthetic fracture, wound dehiscence, blood transfusion, reoperation, hospital-acquired pneumonia, pulmonary embolism, cardiac events, cerebrovascular events, acute renal failure, or in-hospital mortality (Table 5).

**Table 4:** Baseline Patient Characteristics After One-to-One Propensity Score Matching Stratified by Brinkman Index (≥ 600 vs < 600)

	≥ 600	< 600	SMD
n	18506	18506	
Age	65.0 ± 9.3	65.0 ± 11.0	0.007
Gender (%)			
Men	11,115 (60.1)	11,187 (60.5)	0.008
Women	7391 (39.9)	7319 (39.5)	
BMI	24.7 ± 4.1	24.7 ± 4.2	0.010
Uni-rateral THA	18037 (97.5)	18048 (97.5)	0.004
Diagnosis for THA (%)			
OA	14673 (79.3)	14803 (80.0)	0.017
ONFH	3599 (19.4)	3477 (18.8)	
RA	234 (1.3)	226 (1.2)	
CCI	0.63 ± 0.97	0.61 ± 0.97	0.026
Length of hospital stay	25.3 ± 14.8	24.7 ± 14.4	0.042
Cement	3432 (18.6)	3378 (18.3)	0.008
CAOS	5100 (27.6)	5508 (29.8)	0.049
Post-operative Anticoagulate agent	13277 (71.7)	13369 (72.2)	0.011

Age, BMI, CCI, and length of hospital stay are shown as mean ± standard deviation P-values of < 0.001 are considered significant by the Student-t test andχ2 test

BMI: Boby Mass Index, THA; Total Hip Arthroplasty, OA: Osteoarthritis, ONFH; Osteonecrosis of Femoral Head, RA; Rhumatoid Arthritis, CCI; Charlson Comobidity Index, CAOS; Computer Assisted Orthopaedic Surgery SMD; Standardized Mean Difference

**Table 5:** Association Between a Brinkman Index ≥ 600 and Postoperative Complications

Complications			Univariate analysis			Multivariable analysis		
	n	OR	95% CI	P-value	OR	95% CI	χ2 statics	P-value
Dislocation	315	1.205	0.964 to 1.505	0.113	1.042	0.770 to 1.411	0.071	0.790
Infection	386	0.969	0.793 to 1.184	0.798	0.953	0.777 to 1.168	0.214	0.644
Periprosthetic fracture	132	0.913	0.648 to 1.285	0.663	0.854	0.598 to 1.220	0.75	0.386
Wound dehiscence	54	0.8	0.467 to 1.368	0.496	0.784	0.457 to 1.344	0.786	0.375
Transfusion	20868	1.016	0.975 to 1.058	0.470	1.016	0.975 to 1.059	0.585	0.444
Reoperation	615	1.168	0.996 to 1.371	0.061	1.175	0.938 to 1.471	1.983	0.159
Hospital-acquired pneumonia	59	1.566	0.928 to 2.644	0.117	1.611	0.951 to 2.729	3.23	0.072
DVT	1761	0.895	0.813 to 0.985	0.025	0.84	0.813 to 0.985	5.202	0.023
PE	61	0.742	0.447 to 1.234	0.305	0.739	0.444 to 1.229	1.376	0.241
Cardiac event	12	0.714	0.227 to 2.251	0.774	0.794	0.248 to 2.546	0.152	0.697
Cerebrovascular event	64	0.828	0.506 to 1.356	0.532	0.834	0.509 to 1.368	0.519	0.471
Acute renal falure	16	2.2	0.764 to 6.335	0.210	2.225	0.773 to 6.407	2.372	0.124
Mortality during hospitalization	21	0.5	0.202 to 1.239	0.189	0.481	0.198 to 1.205	2.587	0.108

P-values of < 0.001 are considered significant by the χ2 test

OR; Odds Ratio, CI; Confidence Interval, DVT; Deep Vein Thrombosis, PE; Pulmonary Embolism

## Discussion

In this nationwide propensity score–matched study using the Japanese DPC database, smoking was independently associated with an increased risk of postoperative infection following elective THA. In contrast, smoking was not significantly associated with dislocation, periprosthetic fracture, wound dehiscence, reoperation, major medical complications, or in-hospital mortality. Additionally, smoking was associated with a lower likelihood of blood transfusion, and no dose-dependent association was observed when heavy smoking was defined as a Brinkman Index ≥ 600. Notably, this study represents one of the few nationwide analyses specifically evaluating the impact of smoking on perioperative outcomes after elective THA. The Japanese DPC database provides a robust platform for large-scale, nationwide observational studies in orthoapedics and has been widely used to assess perioperative outcomes in hip fracture surgery and arthroplasty [25–28].

The association between smoking and postoperative infection observed in the present study is consistent with prior large-scale observational studies and meta-analyses. Bedard et al. demonstrated in a comprehensive meta-analysis that tobacco use significantly increased the risk of wound complications and periprosthetic joint infection (PJI) after total joint arthroplasty, with current smokers exhibiting the highest risk [14]. Similarly, Bojan et al. reported that smoking was associated with a higher incidence of deep infection and infection-related revision surgery following arthroplasty [15]. More recently, Hadad et al. identified smoking as a risk factor for superficial and deep surgical site infection as well as early readmission after THA [20]. Smoking adversely affects tissue oxygenation, microvascular circulation, and immune function, leading to impaired wound healing and increased susceptibility to infection. These mechanisms provide a strong rationale for smoking cessation as part of perioperative optimization in patients undergoing THA. The magnitude of association observed in our cohort was modest compared with some Western studies [13–15, 20]. This discrepancy may reflect differences in outcome definitions, as the present analysis was limited to in-hospital infections captured in administrative data, whereas many previous studies focused on 30-day or longer-term PJI outcomes. Differences in perioperative management protocols and patient characteristics across healthcare systems may also contribute.

In contrast to infection, smoking was not significantly associated with major medical complications or early reoperation in the present study. This finding differs from reports by Debbi et al., who demonstrated substantially increased risks of inpatient medical complications and mortality among smokers undergoing THA or total knee arthroplasty [4], as well as from the findings of Bongers et al., who reported higher risks of early revision and medical complications in smokers [19]. Variations in study populations, comorbidity profiles, and the time window used to define complications may explain these differences [12]. Importantly, our use of propensity score matching achieved a good balance in measured covariates, which may have attenuated associations observed in less adjusted analyses.

An unexpected finding of the present study was the lower likelihood of blood transfusion among smokers. One possible explanation is the higher baseline haemoglobin levels commonly observed in smokers as a result of chronic hypoxic stimulation of erythropoiesis, which may reduce transfusion requirements despite comparable intraoperative blood loss. Previous studies have explored the association between smoking and perioperative blood transfusion in joint arthroplasty; however, the available evidence remains limited and somewhat inconsistent. Khan et al. reported that smokers undergoing THA were less likely to require blood transfusion than non-smokers in a single-center cohort study, despite a higher overall complication rate among smokers [8]. In contrast, earlier studies investigating predictors of blood loss during THA included smoking history as a covariate in multivariable analyses but did not identify a consistent independent association between smoking and transfusion requirements [29]. Moreover, a recent systematic review and meta-analysis in total knee arthroplasty found no significant difference in the risk of blood transfusion between smokers and non-smokers, suggesting that the relationship between smoking and perioperative blood loss may vary according to surgical procedure and study population [30]. Taken together, these findings indicate that the impact of smoking on transfusion risk in arthroplasty remains incompletely understood. The present nationwide propensity score–matched analysis using the Japanese DPC database adds population-level evidence to this limited literature by demonstrating a lower likelihood of blood transfusion among smokers without a clear dose-dependent relationship. Alternatively, unmeasured factors such as institutional transfusion thresholds, perioperative anemia management strategies, or surgical complexity may have influenced this association. Given the absence of laboratory data in the DPC database, including baseline haemoglobin levels, these results should be interpreted with caution [5, 25–28].

When smoking intensity was assessed using the Brinkman Index, no significant association was observed between heavy smoking (≥ 600) and postoperative complications. Evidence directly comparing complication risk between heavy and moderate smokers in total joint arthroplasty remains limited. In a large single-center cohort including both primary and revision total joint arthroplasty, Ehnert et al. reported a dose-dependent increase in overall complications with greater smoking exposure, with complication rates increasing from non-smokers to moderate smokers (1–20 pack-years) and further to heavy smokers (> 20 pack-years) [31]. Similarly, a systematic review by Singh et al. demonstrated that higher pack-year exposure was associated with an increased risk of systemic complications after THA and TKA in multivariable-adjusted analyses, with patients having very high cumulative exposure (e.g., > 40 pack-years) showing substantially higher odds compared with non-smokers [32]. In contrast, Heckmann et al. reported no significant association between pack-year–defined smoking intensity and wound complications, thromboembolic events, or overall complications following THA [16]. Consistent with these findings, the present nationwide analysis did not identify a clear dose-dependent relationship when heavy smoking was defined as a Brinkman Index ≥ 600. Differences in exposure definitions (pack-years vs Brinkman Index), case mix (primary vs revision procedures), and unmeasured factors such as perioperative management strategies and residual confounding may partly explain these discrepant findings. Moreover, the restriction of our analysis to early in-hospital outcomes may have limited the ability to detect dose-dependent effects that manifest later, including chronic infection or subsequent revision surgery.

The present findings reinforce smoking as an important modifiable risk factor for postoperative infection following THA. Given accumulating evidence that smoking cessation reduces postoperative complication rates [6, 14, 33], structured preoperative smoking cessation programs may play a key role in improving surgical outcomes. In particular, a randomized clinical trial involving patients undergoing elective hip or knee arthroplasty demonstrated that an intensive preoperative smoking intervention significantly reduced overall postoperative complications, especially wound-related events. From a public health perspective, the present results provide population-level evidence supporting perioperative optimization strategies, including smoking cessation, in elective hip arthroplasty.

Several potential limitations of this study should be acknowledged. First, the analysis was restricted to patients who underwent THA at institutions reporting to the Japanese DPC database, thereby excluding procedures performed in non-reporting beds, which account for approximately 30% of general hospital beds in Japan. Second, smoking status was identified using administrative codes and did not allow differentiation between current and former smokers. Third, because the DPC database captures information only during hospitalization, the postoperative observation period was limited, and complications occurring after discharge could not be assessed. In addition, despite rigorous one-to-one propensity score matching, residual confounding due to unmeasured variables—such as operative time, intraoperative blood loss, laboratory data, surgical approach, implant positioning, and postoperative rehabilitation protocols cannot be completely excluded. Finally, the severity of comorbidities and complications could not be evaluated, and events not captured by DPC coding were inherently unmeasurable. Despite these limitations, this study has several notable strengths. Most importantly, it represents a large-scale, nationwide analysis using a comprehensive administrative database, enabling robust evaluation of relatively uncommon postoperative complications with sufficient statistical power. Moreover, this study provides real-world evidence reflecting contemporary clinical practice across Japan, enhancing the generalizability of the findings. To our knowledge, this is one of the few nationwide studies from Asia to comprehensively evaluate the association between smoking and postoperative outcomes following elective THA.

## Conclusion

In this nationwide propensity score–matched study, smoking was associated with an increased risk of early postoperative infection following elective THA. Smoking was not associated with other major complications or in-hospital mortality, and no dose-dependent relationship was observed. These results highlight smoking cessation as an important component of perioperative optimization in THA.

## Data Availability

No datasets were generated or analysed during the current study.

## Abbreviations

THA: Total hip arthroplasty
DPC: Diagnosis Procedure Combination
PS: Propensity score
BMI: Body mass index
CCI: Charlson Comorbidity Index
SMD: Standardized mean difference
OR: Odds ratio
CI: Confidence interval
DVT: Deep vein thrombosis
PE: Pulmonary embolism
OA: Osteoarthritis
ONFH: Osteonecrosis of the femoral head
RA: Rheumatoid arthritis
SD: Standard deviation
PJI: Periprosthetic joint infection
